# Association of short-term glycemic variability with subclinical myocardial injury in hospitalized patients with type 2 diabetes: a retrospective cross-sectional study

**DOI:** 10.3389/fmed.2026.1725076

**Published:** 2026-02-10

**Authors:** Ling Wu, Liying Zhang, Fangli Zhou

**Affiliations:** 1 Department of Endocrinology and Metabolism, West China Hospital, Sichuan University, Chengdu, Sichuan, China; 2 West China School of Nursing, Sichuan University, Chengdu, Sichuan, China; 3 Department of Endocrinology and Metabolism, West China Hospital, Sichuan University, Chengdu, Sichuan, China

**Keywords:** glycemic variability, risk prediction, subclinical myocardial injury, troponin T, type 2 diabetes mellitus

## Abstract

**Background:**

Subclinical myocardial injury (SMI) represents an early, asymptomatic stage of cardiac damage characterized by elevated high-sensitivity cardiac troponin T (hs-cTnT) levels in the absence of overt ischemia. Glycemic variability has been increasingly recognized as a cardiovascular risk factor beyond chronic hyperglycemia, but its relationship with SMI in type 2 diabetes mellitus (T2DM) remains unclear.

**Methods:**

This retrospective cross-sectional analysis included 324 hospitalized patients with T2DM consecutively admitted between January 2021 and December 2023 at a tertiary hospital in Southwest China. SMI was defined as hs-cTnT > 14 ng/L without ischemic symptoms or electrocardiographic abnormalities. Clinical, metabolic, and laboratory data were extracted from electronic medical records. Short-term (in-hospital) glycemic variability was quantified using the standard deviation (SD) and coefficient of variation (CV) of all capillary glucose measurements obtained during hospitalization. Univariate and multivariate logistic regression analyses identified independent predictors of SMI. Model discrimination, calibration, and nomogram-based prediction were evaluated.

**Results:**

Among 324 patients, 128 (39.5%) exhibited SMI. In multivariate analysis, eight variables were independently associated with SMI: age (OR = 1.05, *P* = 0.001), BMI (OR = 1.10, *P* = 0.006), diabetes duration (OR = 1.06, *P* = 0.004), insulin use (OR = 1.72, *P* = 0.015), SD of glucose (OR = 3.11, *P* < 0.001), systolic blood pressure (OR = 1.02, *P* = 0.038), hs-CRP (OR = 1.08, *P* = 0.009), and eGFR (OR = 0.97, *P* = 0.003). The predictive model showed good discrimination (AUC = 0.832, 95% CI 0.787–0.877) and good calibration (Hosmer–Lemeshow *P* = 0.47). A nomogram based on these predictors provided individualized risk estimation with high clinical interpretability.

**Conclusion:**

Short-term (in-hospital) glycemic variability, as reflected by the standard deviation of inpatient glucose readings, was independently associated with subclinical myocardial injury in hospitalized patients with type 2 diabetes. These findings suggest that glycemic variability may serve as a risk marker for subclinical myocardial injury in hospitalized patients with T2DM; however, causal relationships and temporal ordering cannot be inferred from this cross-sectional analysis.

## Introduction

Type 2 diabetes mellitus (T2DM) has emerged as a global health challenge, with its incidence and prevalence escalating rapidly across diverse populations. Recent epidemiological studies highlight that T2DM not only imposes a substantial burden through metabolic dysregulation but also serves as a major contributor to cardiovascular morbidity and mortality worldwide (1). Cardiovascular complications, particularly those affecting the myocardium, account for a significant proportion of adverse outcomes in individuals with T2DM, often manifesting long before overt clinical events. The insidious nature of myocardial involvement in T2DM frequently results in delayed diagnosis and treatment, ultimately compromising patient prognosis and increasing the strain on healthcare systems (2). Against the backdrop of rising T2DM prevalence and its profound impact on global cardiovascular health, there is an urgent need to re-examine the paradigms guiding early detection and risk assessment of cardiac injury in this vulnerable population.

Current epidemiological data underscore the magnitude of the problem: cardiovascular disease remains the leading cause of death among individuals with T2DM, with estimates suggesting that up to two-thirds of diabetic patients will experience a cardiovascular event during their lifetime (3). Moreover, studies indicate that myocardial structure and function may be compromised in diabetic individuals even in the absence of clinical symptoms, with subclinical myocardial injury (SMI) representing a critical, yet under-recognized, stage in the natural history of diabetic heart disease (4, 5). The detection of SMI has been facilitated by advances in non-invasive imaging and the development of sensitive biomarkers such as high-sensitivity cardiac troponins, which can identify early myocardial damage prior to the onset of overt heart failure or ischemic events (6, 7). Nonetheless, the silent progression of myocardial injury in T2DM continues to pose significant diagnostic and therapeutic challenges in routine clinical practice.

In recent years, the scientific community has made considerable efforts to delineate the mechanisms and risk factors underlying SMI in T2DM. Traditional risk stratification tools have largely relied on established cardiovascular risk factors and mean glycemic indices, such as glycated hemoglobin (HbA1c) (8). While HbA1c has utility in predicting macrovascular complications, accumulating evidence suggests that it may not fully capture the dynamic glycemic milieu experienced by patients on a day-to-day basis (9). Emerging data point to the independent and deleterious role of glycemic variability—fluctuations in blood glucose levels over short intervals—in promoting cardiovascular injury through mechanisms involving oxidative stress, inflammation, and endothelial dysfunction (10). Despite these advances, the precise contribution of short-term glycemic variability to the risk of SMI in T2DM remains insufficiently characterized, particularly with respect to its interaction with other metabolic and clinical parameters (11). Furthermore, there is a paucity of validated, user-friendly predictive tools capable of integrating glycemic variability and multidimensional clinical indices to identify individuals at heightened risk for SMI at an early, potentially reversible stage.

This study addresses these critical knowledge gaps by focusing on the independent effect of short-term glycemic variability on subclinical myocardial injury among hospitalized patients with T2DM. Unlike previous investigations that have primarily emphasized mean glycemic control or overt cardiovascular events, the present research adopts a comprehensive approach, leveraging high-sensitivity cardiac troponin T (hs-cTnT) as a sensitive biomarker for the early detection of myocardial injury (12). The novelty of this work lies in its systematic evaluation of the predictive value of glycemic variability—quantified by the standard deviation of inpatient glucose measurements—while simultaneously considering a spectrum of demographic, metabolic, cardiovascular, renal, and inflammatory factors. By elucidating the interplay between glycemic fluctuations and myocardial vulnerability, this study seeks to advance the current understanding of early diabetic cardiomyopathy and establish a foundation for risk-guided intervention.

To achieve this goal, a retrospective cross-sectional design is employed, utilizing real-world data derived from the electronic health records of a tertiary care hospital. The study cohort comprises adult inpatients with T2DM, carefully selected based on stringent inclusion and exclusion criteria to ensure the reliability and generalizability of the findings. Comprehensive data collection encompasses demographic information, diabetes-related variables (including disease duration and treatment modalities), cardiovascular and renal indices, inflammatory markers, and detailed inpatient glycemic profiles. Short-term glycemic variability is operationalized as the standard deviation of capillary blood glucose values recorded throughout the hospitalization period, providing a practical and clinically relevant measure. The primary outcome, subclinical myocardial injury, is defined by elevated hs-cTnT levels in the absence of symptomatic cardiovascular disease, consistent with contemporary biomarker-based definitions (13). Multivariate logistic regression modeling is utilized to identify independent predictors of SMI, with subsequent development of a nomogram for individualized risk estimation.

The overarching objectives of this research are twofold: first, to clarify the role of short-term glycemic variability as an independent risk factor for subclinical myocardial injury in patients with T2DM; and second, to construct and validate a pragmatic predictive tool that integrates glycemic variability with other pertinent clinical variables for early risk stratification. By filling a critical gap in the literature, this study aspires to inform the development of targeted screening and preventive strategies for diabetic patients at risk of occult myocardial injury, thereby enhancing opportunities for timely intervention and improved long-term cardiovascular outcomes.

## Materials and methods

### Study design and setting

This retrospective cross-sectional analysis was conducted at a tertiary hospital in Southwest China that serves as a regional referral center for diabetes management. Electronic medical records were reviewed to extract clinical and laboratory data of hospitalized patients with type 2 diabetes mellitus. The study period extended from January 2021 to December 2023, and all eligible cases admitted for glycemic control or comprehensive metabolic evaluation during this period were consecutively included. In routine practice at our center, these hospitalizations were primarily for diabetes-related management, including glycemic optimization and comprehensive metabolic evaluation, rather than for planned surgery or primary acute cardiac care.

### Participants

Patients were eligible for inclusion if they met the following criteria: (1) diagnosis of type 2 diabetes mellitus (T2DM) according to the 2020 American Diabetes Association criteria; (2) availability of serial glucose monitoring data during hospitalization for calculation of short-term glycemic variability; (3) measurement of high-sensitivity cardiac troponin T (hs-cTnT) on admission. Exclusion criteria were: (1) acute coronary syndrome, acute heart failure exacerbation, myocarditis, or other overt acute cardiac conditions at admission, defined by clinician-documented diagnoses together with compatible ischemic symptoms and/or electrocardiographic evidence of acute ischemia; (2) chronic kidney disease stage ≥ 4 or dialysis dependence; (3) severe infection, systemic inflammatory disease, or malignancy; (4) incomplete key clinical or laboratory data. A total of 324 patients were included after applying the inclusion and exclusion criteria. Among them, 128 (39.5%) met the definition of subclinical myocardial injury (SMI).

### Definitions and variables

Subclinical myocardial injury (SMI) was defined as serum high-sensitivity cardiac troponin T (hs-cTnT) levels exceeding 14 ng/L, corresponding to the 99th percentile upper reference limit, in the absence of ischemic symptoms or electrocardiographic evidence of myocardial infarction. For outcome ascertainment, we used the baseline hs-cTnT value measured at admission (i.e., the first hs-cTnT result available in the electronic medical record for the index hospitalization). Although repeat hs-cTnT testing may be performed when clinically indicated in routine care, serial measurements were not obtained using a standardized protocol across all patients and were therefore not analyzed in this study. The variables evaluated in this study included demographic characteristics [age, sex, body mass index (BMI, kg/m^2^), smoking history, and hypertension], diabetes-related factors [diabetes duration, glycated hemoglobin (HbA1c, %), insulin use, and glycemic variability indices], and cardiovascular as well as renal markers [systolic blood pressure (SBP), low-density lipoprotein cholesterol (LDL-C), estimated glomerular filtration rate (eGFR, mL/min/1.73 m^2^), and high-sensitivity C-reactive protein (hs-CRP)]. Glycemic variability was assessed by calculating the standard deviation (SD) and coefficient of variation (CV) of all capillary glucose readings obtained during hospitalization, with CV defined as SD divided by the mean glucose level multiplied by 100%. All laboratory assays were conducted in the hospital’s central laboratory using standardized procedures and strict quality control protocols to ensure measurement accuracy and comparability across participants.

### Operational definition of short-term glycemic variability

In this study, “short-term” glycemic variability was operationally defined as glucose fluctuations captured during the index hospitalization. Specifically, capillary glucose values were collected from the first bedside glucometer measurement recorded after admission to the last bedside glucometer measurement recorded before discharge, and all available inpatient capillary glucose readings within this time window were used to compute glycemic variability metrics (SD and CV). Bedside glucose monitoring was performed as part of routine inpatient care, typically 5–7 measurements per day (e.g., pre-meal and bedtime measurements, with additional checks as clinically indicated).

### Data collection and measurement

Clinical and laboratory data were extracted retrospectively from the hospital’s electronic medical record system. The primary reason for hospitalization (primary admission diagnosis) for the index admission was extracted from the electronic medical record based on admission notes and discharge diagnosis coding. Admission diagnoses were categorized into clinically meaningful groups and are summarized in Supplementary Table S2. All information, including demographic characteristics, comorbidities, and medication history, was verified by two independent investigators to ensure accuracy. Blood pressure values recorded at admission were obtained from routine nursing assessments performed in the seated position after at least 5 min of rest. Laboratory parameters, including fasting plasma glucose, glycated hemoglobin (HbA1c), lipid profile, high-sensitivity C-reactive protein (hs-CRP), and high-sensitivity cardiac troponin T (hs-cTnT), were retrieved from standardized biochemical test reports performed by the hospital’s central laboratory. The hs-cTnT value used to define SMI was the admission (baseline) measurement, retrieved from the central laboratory report in the electronic medical record. Serum creatinine levels were used to calculate the estimated glomerular filtration rate (eGFR) using the CKD-EPI equation.

Glycemic variability parameters were calculated based on all capillary glucose values recorded during hospitalization through routine bedside glucometer monitoring (typically 5–7 measurements per day). To maintain data quality, only glucose records with complete time stamps and within-instrument calibration were included. All measurements had been performed using standardized instruments and reagents as part of the hospital’s routine quality-controlled laboratory workflow. Before statistical analysis, data integrity and plausibility were carefully verified and cross-checked to minimize recording or extraction errors. To enhance transparency regarding sampling intensity and monitoring duration, we summarized the number of inpatient capillary glucose measurements per patient [median (IQR), range] and the length of stay [median (IQR), range] for the study cohort and reported these descriptive statistics in the Results.

### Bias control

To minimize potential bias, all eligible patients meeting predefined criteria were consecutively enrolled. Laboratory and clinical data were collected prospectively during hospitalization and retrospectively reviewed by blinded investigators. Patients with acute cardiovascular events or missing data were excluded to avoid misclassification of myocardial injury. Multicollinearity among covariates was assessed using the variance inflation factor (VIF); when high collinearity was detected (e.g., between SD and CV of glucose), only one variable (SD) was retained in the multivariate model.

### Sample size considerations

All eligible patients during the study period were included to maximize statistical power. *Post-hoc* estimation based on the final model (8 predictors, outcome prevalence 39.5%) indicated that the sample size of 324 provided adequate power (>0.9) to detect odds ratios ≥ 1.5 at a two-sided α = 0.05, satisfying the rule of at least 10 outcome events per variable.

### Statistical analysis

Continuous variables were tested for normality using the Shapiro–Wilk test. Normally distributed variables were expressed as mean ± standard deviation (SD) and compared using the independent-samples *t*-test; non-normally distributed variables were reported as median [interquartile range (IQR)] and compared using the Mann–Whitney U test. Categorical variables were presented as counts (percentages) and compared using the χ^2^ test or Fisher’s exact test, as appropriate.

Univariate logistic regression was first performed to identify factors associated with SMI. Variables with *P* < 0.05 in univariate analysis or those of clinical relevance were entered into a multivariate logistic regression model using the enter method to identify independent predictors. Multicollinearity was assessed via VIF, and model fit was evaluated using the Hosmer–Lemeshow goodness-of-fit test.

The discriminative ability of the model was assessed by the area under the receiver operating characteristic (ROC) curve (AUC), with 95% confidence intervals obtained by bootstrapping (1,000 iterations). Calibration was examined using calibration plots comparing predicted and observed probabilities. Model performance metrics—including sensitivity, specificity, positive predictive value (PPV), negative predictive value (NPV), accuracy, and Youden Index—were derived at the optimal probability threshold determined by the Youden Index method.

A nomogram was constructed based on the final multivariate model to enable individualized risk prediction of SMI. All statistical analyses were performed using R software (version 4.3.3, R Foundation for Statistical Computing, Vienna, Austria), with *P* < 0.05 considered statistically significant.

## Results

### Baseline characteristics of the study population

As summarized in Table 1, a total of 324 hospitalized patients with type 2 diabetes mellitus (T2DM) were included in the analysis, among whom 128 (39.5%) were identified with subclinical myocardial injury (SMI), defined as elevated high-sensitivity cardiac troponin T (hs-cTnT) levels > 14 ng/L in the absence of ischemic symptoms. Patients with SMI were significantly older than those without myocardial injury (66.5 ± 10.2 vs. 60.3 ± 9.6 years, *P* < 0.001) and had a higher body mass index (26.7 ± 3.5 vs. 25.2 ± 3.3 kg/m^2^, *P* = 0.004). The prevalence of smoking (38.3% vs. 25.0%, *P* = 0.013) and hypertension (71.1% vs. 59.2%, *P* = 0.032) was also higher among patients with SMI. Regarding diabetes-related characteristics, individuals with SMI exhibited a longer median duration of diabetes (11.0 vs. 6.0 years, *P* < 0.001), higher HbA1c levels (8.4 ± 1.7 vs. 7.9 ± 1.4%, *P* = 0.002), and were more frequently treated with insulin (53.1% vs. 35.2%, *P* = 0.002), reflecting poorer glycemic control and more advanced disease course. Indices of glycemic variability were markedly elevated in the SMI group, including both the standard deviation (2.5 ± 0.6 vs. 1.8 ± 0.5 mmoL/L, *P* < 0.001) and coefficient of variation of glucose (25.5% vs. 20.1%, *P* < 0.001). In terms of cardiovascular and renal parameters, patients with SMI had higher systolic blood pressure (142.7 ± 18.0 vs. 134.2 ± 17.5 mmHg, *P* = 0.001), slightly higher LDL-C levels (2.8 ± 0.9 vs. 2.6 ± 0.7 mmoL/L, *P* = 0.039), and significantly lower estimated glomerular filtration rate (72.1 ± 19.1 vs. 82.5 ± 15.3 mL/min/1.73 m^2^, *P* < 0.001). Inflammatory activity was also greater in the SMI group, as indicated by elevated high-sensitivity C-reactive protein [median 4.7 mg/L (2.1–7.5) vs. 2.8 mg/L (1.2–5.4), *P* < 0.001]. Regarding inpatient monitoring intensity, the median number of capillary glucose measurements per patient was 32 (IQR 24–41, range 18–56). The median length of stay was 6 days (IQR 4–8, range 3–12). These distributions are provided to contextualize the sampling window underpinning the calculation of short-term glycemic variability. The primary admission diagnoses for the index hospitalization are summarized in Supplementary Table S2 to contextualize the clinical setting in which in-hospital glucose monitoring and hs-cTnT assessment were performed.

**TABLE 1:** Baseline characteristics of patients with and without subclinical myocardial injury (*n* = 324).

Variables	Total (*n* = 324)	No injury (*n* = 196)	Subclinical injury (*n* = 128)	*P*-value
**Demographics**				
Age, years (mean ± SD)	62.7 ± 10.4	60.3 ± 9.6	66.5 ± 10.2	< 0.001
Male sex, n (%)	182 (56.2)	103 (52.6)	79 (61.7)	0.132
BMI, kg/m^2^ (mean ± SD)	25.8 ± 3.4	25.2 ± 3.3	26.7 ± 3.5	0.004
Smoking history, n (%)	98 (30.2)	49 (25.0)	49 (38.3)	0.013
Hypertension, n (%)	207 (63.9)	116 (59.2)	91 (71.1)	0.032
**Diabetes-related indicators**				
Diabetes duration, years [median (IQR)]	8.0 [4.0–13.0]	6.0 [3.0–10.0]	11.0 [6.0–16.0]	< 0.001
HbA1c, % (mean ± SD)	8.1 ± 1.6	7.9 ± 1.4	8.4 ± 1.7	0.002
Insulin use, n (%)	137 (42.3)	69 (35.2)	68 (53.1)	0.002
**Glycemic variability**				
SD of glucose, mmoL/L (mean ± SD)	2.1 ± 0.6	1.8 ± 0.5	2.5 ± 0.6	< 0.001
CV of glucose, % (mean ± SD)	22.4 ± 5.8	20.1 ± 5.2	25.5 ± 5.3	< 0.001
**Cardiovascular and renal markers**				
SBP, mmHg (mean ± SD)	137.6 ± 18.2	134.2 ± 17.5	142.7 ± 18.0	0.001
LDL-C, mmoL/L (mean ± SD)	2.7 ± 0.8	2.6 ± 0.7	2.8 ± 0.9	0.039
eGFR, mL/min/1.73 m^2^ (mean ± SD)	78.2 ± 17.6	82.5 ± 15.3	72.1 ± 19.1	< 0.001
hs-CRP, mg/L [median (IQR)]	3.4 [1.5–6.7]	2.8 [1.2–5.4]	4.7 [2.1–7.5]	< 0.001
hs-cTnT, ng/L (mean ± SD)	10.6 ± 6.2	6.8 ± 2.9	16.1 ± 5.3	< 0.001

BMI, body mass index; HbA1c, glycated hemoglobin; SD, standard deviation of glucose levels measured during hospitalization; CV, coefficient of variation of glucose (SD/mean × 100%); SBP, systolic blood pressure; LDL-C, low-density lipoprotein cholesterol; eGFR, estimated glomerular filtration rate; hs-CRP, high-sensitivity C-reactive protein; hs-cTnT, high-sensitivity cardiac troponin T. Subclinical myocardial injury was defined as hs-cTnT levels > 14 ng/L (99th percentile upper reference limit) without clinical symptoms of myocardial infarction.

### Univariate logistic regression analysis

Univariate logistic regression was conducted to identify potential factors associated with subclinical myocardial injury (SMI) in patients with type 2 diabetes mellitus. As shown in Table 2, several demographic, metabolic, and cardiovascular variables demonstrated significant associations with SMI. Increasing age was positively associated with the likelihood of myocardial injury (OR = 1.06, 95% CI: 1.03–1.09, *P* < 0.001), while male sex was not statistically significant (*P* = 0.121). Higher body mass index (BMI) was associated with greater risk (OR = 1.12, 95% CI: 1.04–1.21, *P* = 0.003), as were smoking history (OR = 1.87, 95% CI: 1.17–2.98, *P* = 0.009) and hypertension (OR = 1.74, 95% CI: 1.10–2.76, *P* = 0.017). Diabetes-related variables were also strongly linked to myocardial injury. Longer diabetes duration (OR = 1.08, 95% CI: 1.04–1.12, *P* < 0.001), higher HbA1c levels (OR = 1.29, 95% CI: 1.08–1.55, *P* = 0.006), and insulin use (OR = 2.09, 95% CI: 1.34–3.26, *P* = 0.001) were each associated with increased odds of SMI. Measures of short-term glycemic variability exhibited particularly strong relationships: In terms of cardiovascular and renal factors, higher systolic blood pressure (OR = 1.03, 95% CI: 1.01–1.05, *P* = 0.002), elevated LDL-C (OR = 1.31, 95% CI: 1.02–1.69, *P* = 0.037), and increased high-sensitivity C-reactive protein (hs-CRP) levels (OR = 1.10, 95% CI: 1.05–1.16, *P* < 0.001) were significantly associated with SMI. Conversely, a lower estimated glomerular filtration rate (eGFR) was inversely correlated with myocardial injury (OR = 0.96, 95% CI: 0.94–0.98, *P* < 0.001), indicating that declining renal function may contribute to subclinical myocardial damage in diabetic patients. Collectively, these results suggest that both traditional cardiovascular risk factors and diabetes-related metabolic derangements, particularly glycemic variability, play important roles in the development of subclinical myocardial injury (Table 2).

**TABLE 2:** Univariate logistic regression analysis of factors associated with subclinical myocardial injury in patients with type 2 diabetes mellitus (*n* = 324).

Variables	OR (95% CI)	*P*-value
Age (per 1-year increase)	1.06 (1.03–1.09)	< 0.001
Male sex (vs. female)	1.43 (0.91–2.25)	0.121
BMI (per 1 kg/m^2^ increase)	1.12 (1.04–1.21)	0.003
Smoking history (yes vs. no)	1.87 (1.17–2.98)	0.009
Hypertension (yes vs. no)	1.74 (1.10–2.76)	0.017
Diabetes duration (per 1-year increase)	1.08 (1.04–1.12)	< 0.001
HbA1c (per 1% increase)	1.29 (1.08–1.55)	0.006
Insulin use (yes vs. no)	2.09 (1.34–3.26)	0.001
SD of glucose (per 1 mmoL/L increase)	3.52 (2.43–5.11)	< 0.001
CV of glucose (per 1% increase)	1.18 (1.12–1.25)	< 0.001
SBP (per 1 mmHg increase)	1.03 (1.01–1.05)	0.002
LDL-C (per 1 mmoL/L increase)	1.31 (1.02–1.69)	0.037
eGFR (per 1 mL/min/1.73 m^2^ increase)	0.96 (0.94–0.98)	< 0.001
hs-CRP (per 1 mg/L increase)	1.10 (1.05–1.16)	< 0.001

OR, odds ratio; CI, confidence interval; BMI, body mass index; HbA1c, glycated hemoglobin; SD, standard deviation of inpatient capillary glucose measurements; CV, coefficient of variation of glucose (SD/mean × 100%); SBP, systolic blood pressure; LDL-C, low-density lipoprotein cholesterol; eGFR, estimated glomerular filtration rate; hs-CRP, high-sensitivity C-reactive protein. Subclinical myocardial injury was defined as hs-cTnT > 14 ng/L (99th percentile upper reference limit) in the absence of ischemic symptoms or electrocardiographic evidence of myocardial infarction. Odds ratios for continuous variables are reported per one-unit increase in the corresponding measurement. For categorical variables, the reference group was female (for sex) or no (for smoking, hypertension, and insulin use). Variables with *P* < 0.05 were considered for entry into the multivariate logistic regression model.

### Multivariate logistic regression analysis

Multivariate logistic regression analysis was conducted to identify independent predictors of subclinical myocardial injury (SMI) among patients with type 2 diabetes mellitus (T2DM), adjusting for variables with *P* < 0.05 in the univariate analysis. As summarized in Table 3, eight factors remained significantly associated with the presence of SMI. Among demographic and metabolic parameters, older age (adjusted OR = 1.05, 95% CI: 1.02–1.08, *P* = 0.001) and higher body mass index (BMI) (adjusted OR = 1.10, 95% CI: 1.03–1.19, *P* = 0.006) were independently associated with increased odds of myocardial injury. In addition, longer diabetes duration (adjusted OR = 1.06, 95% CI: 1.02–1.10, *P* = 0.004) and insulin use (adjusted OR = 1.72, 95% CI: 1.11–2.68, *P* = 0.015) were significant predictors, suggesting that both disease chronicity and intensive treatment requirement reflect greater cardiovascular vulnerability. Of note, the standard deviation (SD) of glucose (per 1 mmoL/L increase), representing short-term glycemic variability, showed the largest magnitude of independent association with subclinical myocardial injury in the multivariate model (adjusted OR = 3.11, 95% CI: 2.11–4.60, *P* < 0.001). Each 1 mmoL/L increase in the standard deviation (SD) of glucose was associated with a more than threefold increase in the odds of subclinical myocardial injury, underscoring the strong association between glycemic fluctuations and myocardial vulnerability beyond mean glucose levels. Furthermore, higher systolic blood pressure (SBP) (adjusted OR = 1.02, 95% CI: 1.00–1.04, *P* = 0.038) and elevated high-sensitivity C-reactive protein (hs-CRP) (adjusted OR = 1.08, 95% CI: 1.02–1.14, *P* = 0.009) were independently associated with increased risk, whereas lower estimated glomerular filtration rate (eGFR) (adjusted OR = 0.97, 95% CI: 0.95–0.99, *P* = 0.003) was inversely related to myocardial injury. Collectively, these findings indicate that aging, adiposity, prolonged diabetes duration, insulin dependence, blood pressure elevation, renal dysfunction, systemic inflammation, and particularly glycemic variability are key determinants of subclinical myocardial injury in hospitalized T2DM patients (Table 3).

**TABLE 3:** Multivariate logistic regression analysis of independent predictors of subclinical myocardial injury in patients with type 2 diabetes mellitus (*n* = 324).

Variables	Adjusted OR (95% CI)	*P*-value
Age (per 1-year increase)	1.05 (1.02–1.08)	0.001
BMI (per 1 kg/m^2^ increase)	1.10 (1.03–1.19)	0.006
Diabetes duration (per 1-year increase)	1.06 (1.02–1.10)	0.004
Insulin use (yes vs. no)	1.72 (1.11–2.68)	0.015
SD of glucose (per 1 mmoL/L increase)	3.11 (2.11–4.60)	< 0.001
SBP (per 1 mmHg increase)	1.02 (1.00–1.04)	0.038
eGFR (per 1 mL/min/1.73 m^2^ increase)	0.97 (0.95–0.99)	0.003
hs-CRP (per 1 mg/L increase)	1.08 (1.02–1.14)	0.009

Adjusted OR, adjusted odds ratio; CI, confidence interval; BMI, body mass index; SD, standard deviation of inpatient glucose measurements; SBP, systolic blood pressure; eGFR, estimated glomerular filtration rate; hs-CRP, high-sensitivity C-reactive protein. Subclinical myocardial injury was defined as hs-cTnT > 14 ng/L without clinical or electrocardiographic evidence of myocardial infarction. Odds ratios for continuous variables are reported per one-unit increase in the corresponding measurement. Variables were selected for inclusion based on univariate *P* < 0.05 and clinical relevance. The coefficient of variation (CV) of glucose was excluded from the final model because of multicollinearity with the standard deviation (SD) of glucose.

### Model performance, calibration, and nomogram construction

**FIGURE 1:**
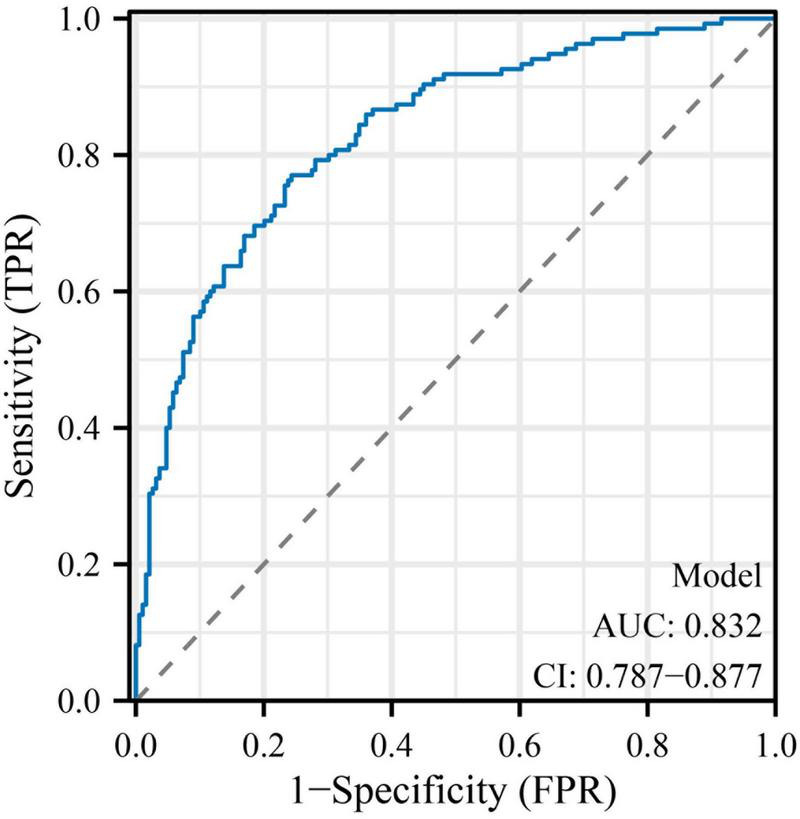
Receiver operating characteristic (ROC) curve for the prediction model of subclinical myocardial injury in patients with type 2 diabetes mellitus. The receiver operating characteristic (ROC) curve demonstrates the discriminative performance of the final multivariate logistic regression model for predicting subclinical myocardial injury (SMI) in hospitalized patients with type 2 diabetes mellitus (T2DM). The area under the curve (AUC) was 0.832 (95% CI: 0.787–0.877), indicating good model discrimination. The optimal probability threshold determined by the Youden Index (cut-off = - 0.5759) achieved a sensitivity of 77.0% and a specificity of 75.7%, reflecting balanced predictive accuracy between true-positive and true-negative classifications.

**FIGURE 2:**
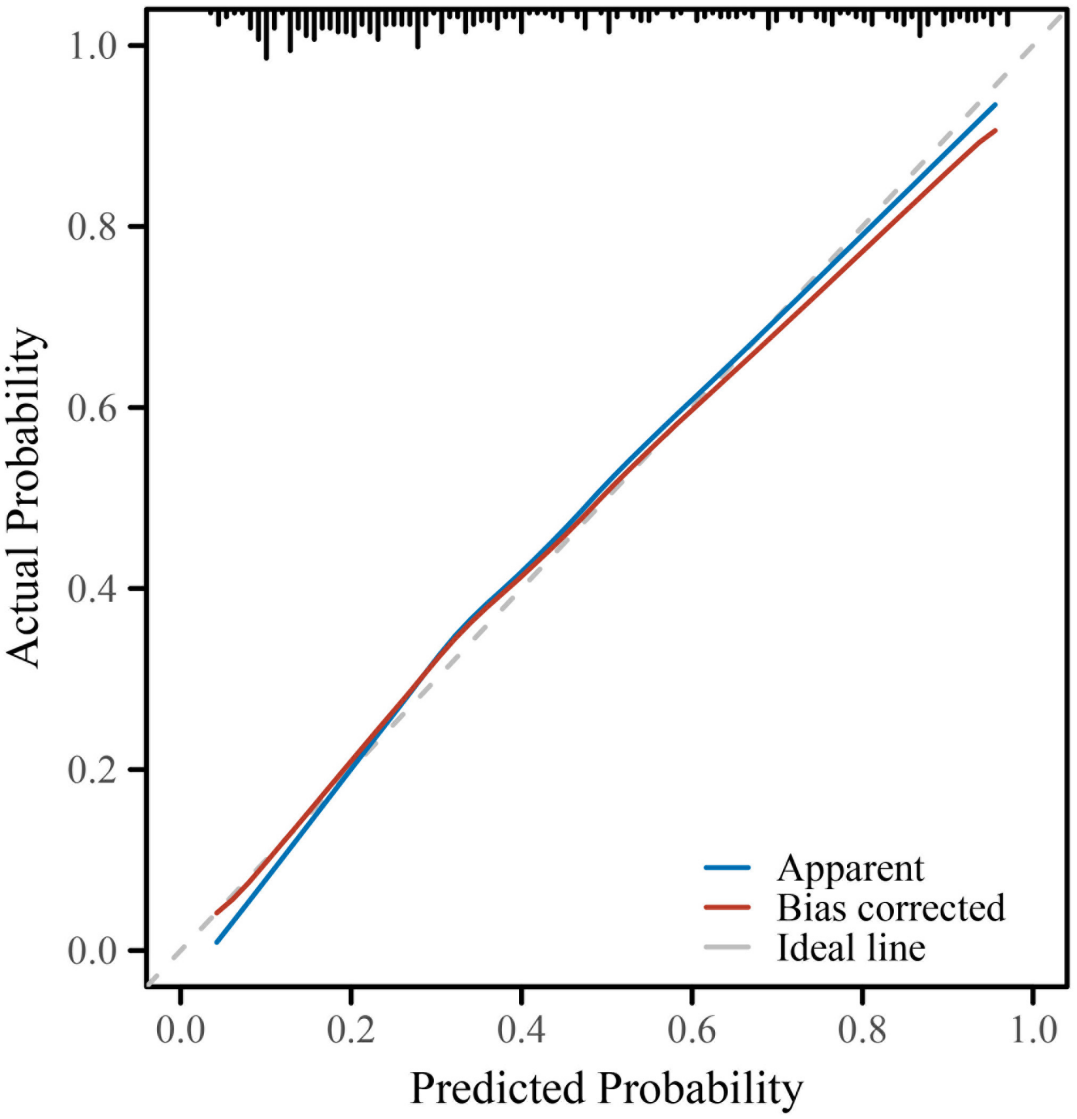
Calibration curve for the multivariate logistic regression model predicting subclinical myocardial injury. Calibration performance of the final predictive model was evaluated using a calibration plot with 1,000 bootstrap resamples. The blue line represents the apparent performance of the model, while the red line shows the bias-corrected performance after internal validation. The dashed gray line indicates the ideal reference (perfect calibration). The close alignment between the bias-corrected and ideal lines demonstrates good agreement between predicted and observed probabilities, confirming that the model was well-calibrated with minimal overfitting (Hosmer–Lemeshow test, *P* = 0.47).

**FIGURE 3:**
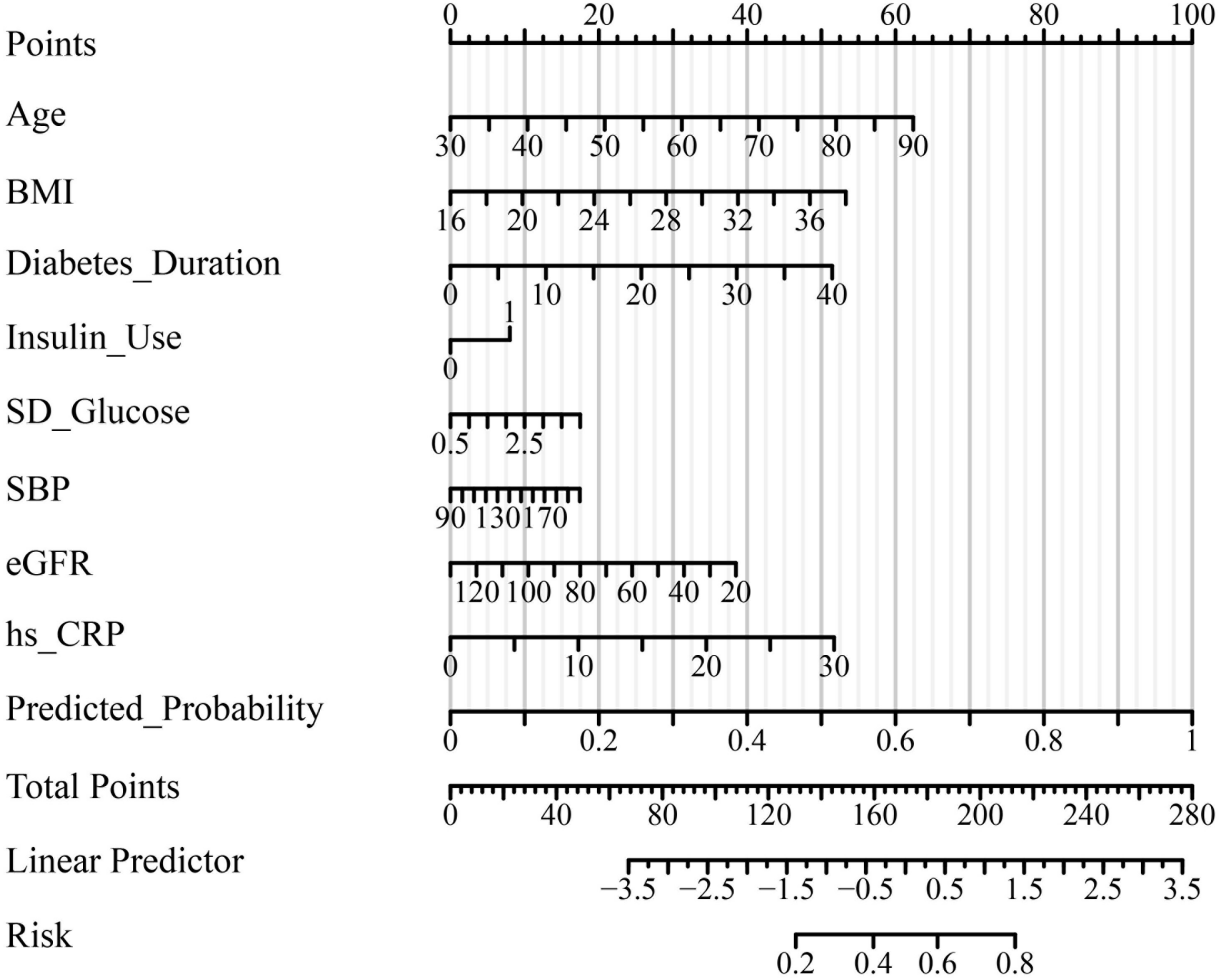
Nomogram for predicting the probability of subclinical myocardial injury in patients with type 2 diabetes mellitus. A nomogram was constructed based on the final multivariate logistic regression model to facilitate individualized risk estimation for subclinical myocardial injury (SMI) in patients with T2DM. The nomogram integrates eight independent predictors: Age, body mass index (BMI), diabetes duration, insulin use, standard deviation (SD) of glucose, systolic blood pressure (SBP), estimated glomerular filtration rate (eGFR), and high-sensitivity C-reactive protein (hs-CRP). Each variable corresponds to a specific point value on the upper scale, and the total points accumulated correspond to a predicted probability of SMI on the lower axis. Higher total scores indicate an increased likelihood of subclinical myocardial injury, allowing intuitive visual assessment of individualized risk.

The final multivariate logistic regression model demonstrated good discriminative ability for predicting subclinical myocardial injury (SMI) in hospitalized patients with type 2 diabetes mellitus (T2DM). As shown in Table 4 and Figure 1, the area under the receiver operating characteristic (ROC) curve (AUC) was 0.832 (95% CI: 0.787–0.877), indicating good discrimination. Clinically, this level of discrimination suggests that the model can reasonably distinguish hospitalized patients who have SMI from those who do not, thereby supporting risk enrichment in settings where hs-cTnT testing is available but downstream cardiac evaluation resources may be limited. At the Youden-derived threshold, the corresponding sensitivity (77.0%) and specificity (75.7%), together with the observed NPV (82.2%), indicate that the model may be most useful for identifying patients at relatively lower predicted risk who are unlikely to have SMI within this inpatient T2DM cohort, while not replacing biomarker-based ascertainment. The optimal cut-off probability of −0.5759 yielded a sensitivity of 77.0% and a specificity of 75.7%, with an overall accuracy of 76.2%. The corresponding positive predictive value (PPV) and negative predictive value (NPV) were 69.3 and 82.2%, respectively, and the Youden Index was 0.527, reflecting a balanced predictive performance. The calibration of the model was assessed using the Hosmer–Lemeshow goodness-of-fit test and a calibration plot (Figure 2). The Hosmer–Lemeshow *P*-value of 0.47 indicated good agreement between predicted and observed probabilities, and the bias-corrected calibration curve closely overlapped with the apparent curve, suggesting minimal overfitting and good model stability. From a clinical perspective, the favorable calibration implies that the predicted probabilities are reasonably aligned with the observed event rates in this study setting, which is important if the nomogram is used to communicate individualized risk estimates to clinicians and patients during hospitalization. To enhance clinical applicability, a nomogram incorporating eight independent predictors—age, body mass index (BMI), diabetes duration, insulin use, standard deviation (SD) of glucose, systolic blood pressure (SBP), estimated glomerular filtration rate (eGFR), and high-sensitivity C-reactive protein (hs-CRP)—was constructed based on the final logistic regression model (Figure 3). For illustration, consider a hospitalized patient with T2DM aged 66 years, BMI 27 kg/m^2^, diabetes duration 11 years, insulin use (yes), SD of inpatient glucose 2.5 mmoL/L, SBP 143 mmHg, eGFR 72 mL/min/1.73 m^2^, and hs-CRP 4.7 mg/L. Using the nomogram (Figure 3), each predictor is mapped to its corresponding point value on the top scale; the points are then summed to obtain a total score, which is subsequently translated to an estimated probability of SMI on the bottom axis. This worked example is intended to demonstrate the calculation procedure for individualized risk estimation within the study setting and does not imply causal effects or intervention benefit. In this study, the nomogram is intended to support risk enrichment and individualized probability estimation within the inpatient T2DM population, rather than serving as a screening tool or a basis for clinical decision-making. In practice, patients with higher predicted risk may warrant closer cardiovascular assessment (e.g., review for non-ischemic causes of hs-cTnT elevation, consideration of repeat troponin testing when clinically indicated, and/or targeted evaluation of blood pressure, renal function, and inflammatory status), while acknowledging that the model does not establish causality. Each variable corresponds to a specific point value, and the total point score predicts the individual probability of subclinical myocardial injury. Higher total scores corresponded to an increased predicted risk, allowing intuitive, individualized risk estimation. Collectively, these results confirm that the model exhibits good discrimination, good calibration, and practical interpretability, providing an interpretable approach to estimate the probability of subclinical myocardial injury in the study setting (Table 4 and Figures 1–3).

**TABLE 4:** Predictive performance of the final multivariate logistic regression model for subclinical myocardial injury in patients with type 2 diabetes (*n* = 324).

Performance metric	Value (95% CI)
Area under the ROC curve (AUC)	0.832 (0.787–0.877)
Optimal cut-off probability	−0.5759
Sensitivity	77.0 %
Specificity	75.7 %
Overall accuracy	76.2 %
True positives (TP)	104
True negatives (TN)	143
False positives (FP)	46
False negatives (FN)	31
Positive predictive value (PPV)	69.3 %
Negative predictive value (NPV)	82.2 %
Youden index	0.527
Hosmer–lemeshow goodness-of-fit test (*P*-value)	0.47

AUC, area under the receiver operating characteristic curve; PPV, positive predictive value; NPV, negative predictive value; CI, confidence interval. The model demonstrated good discrimination for identifying subclinical myocardial injury in hospitalized patients with type 2 diabetes, achieving an AUC of 0.832 with balanced sensitivity and specificity at the optimal probability threshold (−0.5759).

## Discussion

Type 2 diabetes mellitus (T2DM) constitutes a significant global health challenge, primarily due to its profound impact on cardiovascular morbidity and mortality. The disease fosters a milieu conducive to cardiovascular complications, often manifesting initially as subclinical myocardial injury, which precedes overt clinical events such as heart failure and myocardial infarction. This early myocardial damage, frequently undetected by conventional clinical assessments, undermines cardiac function and portends adverse outcomes. Despite advances in glycemic control and cardiovascular risk management, there remains a critical gap in the identification and intervention of these asymptomatic myocardial alterations within the diabetic population. Consequently, delineating the mechanisms and predictors of subclinical myocardial injury in T2DM is imperative to improve early detection strategies and mitigate the progression to clinically manifest cardiovascular disease.

In this context, the present study undertook a comprehensive evaluation of hospitalized patients with T2DM to elucidate the relationship between short-term glycemic variability and subclinical myocardial injury, as indicated by elevated high-sensitivity cardiac troponin T (hs-cTnT). By leveraging multidimensional clinical and laboratory data alongside good statistical modeling, the investigation identified glycemic fluctuations as a key factor independently associated with subclinical myocardial injury interacting with established risk factors including age, body mass index, diabetes duration, insulin therapy, hypertension, inflammatory status, and renal function. The development of a predictive nomogram for individualized risk assessment underscores the translational potential of these findings, setting the stage for targeted interventions aimed at the early cardiovascular protection in T2DM patients. Importantly, the present study was designed to evaluate these associations within a hospitalized T2DM cohort and does not test whether the observed relationships are stronger in diabetes than in non-diabetic populations. Accordingly, the added value of our work lies in population-specific confirmation in a clinically relevant T2DM inpatient setting rather than demonstration of diabetes-specific effect modification.

The observed associations of advanced age, higher body mass index, longer diabetes duration, insulin use, hypertension, systemic inflammation, and renal dysfunction with subclinical myocardial injury in hospitalized patients with type 2 diabetes mellitus are biologically plausible and supported by converging mechanistic evidence. Aging has been shown to increase myocardial susceptibility through cumulative oxidative stress, impaired cellular repair capacity, and progressive microvascular dysfunction, while obesity and hypertension jointly promote chronic low-grade inflammation, endothelial dysfunction, and structural microvascular injury (14–16). A longer duration of diabetes and the requirement for insulin therapy likely reflect cumulative metabolic burden and greater disease severity, characterized by persistent insulin resistance, β-cell dysfunction, and sustained activation of inflammatory and oxidative pathways (17–19). In parallel, reduced estimated glomerular filtration rate and elevated high-sensitivity C-reactive protein underscore the contribution of renal–cardiac crosstalk, neurohormonal activation, and systemic inflammation to myocardial microdamage (20–22). Collectively, these interrelated metabolic, inflammatory, and hemodynamic disturbances delineate a coherent pathophysiological framework for subclinical myocardial injury in type 2 diabetes, consistent with prior epidemiological observations and mechanistic studies (23–25).

Mechanistically, glycemic oscillations may exacerbate subclinical myocardial injury by amplifying oxidative stress and inflammatory activation beyond the effects of sustained hyperglycemia alone (26). Acute glucose fluctuations promote mitochondrial reactive oxygen species generation and endo thelial dysfunction, thereby accelerating myocardial microdamage. The concomitant elevation of hs-CRP in our cohort supports the central role of systemic inflammation in this process, as CRP reflects and propagates vascular and myocardial inflammatory responses (27). Consistent with prior studies, short-term glycemic variability appears more strongly associated with cardiovascular events and subclinical myocardial injury than mean glycemic indices, underscoring the pathophysiological relevance of dynamic glucose excursions in T2DM (28). By restricting the analysis to hospitalized patients with T2DM, our findings provide context-specific evidence in a population characterized by chronic metabolic dysregulation, frequent inpatient glucose monitoring, and a high burden of cardiometabolic comorbidity. This setting may differ from community-based mixed cohorts in measurement intensity and clinical risk profiles, and therefore warrants dedicated evaluation. An additional consideration is the relationship between short-term glycemic variability and HbA1c. HbA1c primarily reflects longer-term average glycemic exposure over the preceding weeks to months, whereas in-hospital variability metrics (e.g., SD and CV) capture short-term glucose excursions and instability during the index admission. These constructs are related but not interchangeable: patients with similar HbA1c may experience markedly different degrees of acute glycemic fluctuation depending on intercurrent illness, nutritional intake, treatment intensification, and counter-regulatory stress responses. From a clinical standpoint, this distinction is relevant because HbA1c alone may not fully reflect short-term dysglycemia during hospitalization, while variability measures can complement HbA1c by identifying patients with acute glucose instability who may be at higher risk of end-organ vulnerability. In this study, HbA1c was associated with SMI in univariate analysis but was not retained as an independent predictor in the final multivariable model, whereas SD of glucose remained significant, suggesting that short-term variability may provide additional risk information within this inpatient setting. Nevertheless, because mean inpatient glucose over the same monitoring window was not included in the multivariable models, we could not fully disentangle the contribution of variability from overall glycemic level, and the incremental predictive value beyond HbA1c should be quantified in future studies using standardized protocols and, ideally, CGM-derived metrics.

The predictive model integrating eight independent risk factors demonstrated good discrimination (AUC 0.832) and calibration, with sustained performance across sensitivity and specificity metrics. The scientific rationale for multi-factorial modeling lies in the recognition that SMI in T2DM arises from the confluence of diverse pathophysiological domains, including metabolic dysregulation, inflammation, hemodynamic stress, and renal impairment, each contributing incrementally to myocardial vulnerability. The application of a nomogram facilitates individualized risk estimation by quantitatively weighing these interdependent contributors, a methodological advance over traditional risk stratification tools that often neglect nuanced interactions among variables (29). However, because model discrimination and calibration were evaluated for the full multivariable model, and predictors were not standardized, the present analysis does not support direct comparison of odds ratio magnitudes across variables measured on different scales, nor does it quantify variable-specific predictive dominance. Comparative analysis with established SMI and cardiovascular risk models reveals that the inclusion of dynamic glycemic variability and inflammation markers such as hs-CRP enhances model performance, consistent with accumulating evidence that metabolic and inflammatory perturbations are critical for early detection of occult myocardial injury (30). Notably, validation and external calibration remain essential for broader clinical application, as model performance can be influenced by population-specific factors, measurement frequency, and endpoint definitions. This underscores the necessity for ongoing refinement and prospective validation to optimize predictive accuracy in diverse T2DM cohorts.

The association between subclinical myocardial injury and multifaceted metabolic disturbances in T2DM reflects a complex interplay of converging pathobiological processes. Glycemic variability, chronic inflammation, hypertension, and renal dysfunction act through partially overlapping molecular pathways that ultimately converge on endothelial injury, microvascular rarefaction, and myocardial fibrosis (31). Glucose fluctuations promote oxidative stress and mitochondrial dysfunction, while persistent inflammation and hemodynamic stress exacerbate microvascular injury and subclinical ischemia; renal impairment further amplifies these effects via neurohormonal and metabolic dysregulation (32). Emerging multi-omics evidence suggests that alterations in lipid metabolism, gut microbiota, and protein homeostasis add additional layers to this systems-level interaction (33). Experimental models support the concept that these mechanisms potentiate one another rather than acting in isolation, linking metabolic stress to progressive myocardial dysfunction through integrated oxi-inflamm-aging and cellular senescence pathways (34). Taken together, these findings suggest that short-term glycemic variability reflects a convergence of metabolic instability, inflammatory activation, and end-organ vulnerability in hospitalized patients with T2DM, while also highlighting the need for multi-target preventive strategies and underscoring several critical unanswered questions regarding causality, temporal dynamics, and modifiability.

Several important limitations should be acknowledged in this study. First, the retrospective cross-sectional design and reliance on single-center data from a tertiary referral hospital may introduce selection bias, as the included population predominantly comprised hospitalized patients with more advanced or poorly controlled diabetes, potentially limiting the generalizability of the findings to broader outpatient or community-based T2DM cohorts. In addition, because the study was conducted at a single center in China, caution is warranted when extrapolating these findings to non-Chinese populations with different healthcare systems, genetic backgrounds, and patterns of inpatient glucose monitoring. Although we excluded overt acute cardiac events and severe systemic conditions, residual confounding from unmeasured factors cannot be fully excluded, including pre-admission medication adherence, outpatient glycemic management intensity, and stress hyperglycemia related to acute illness or physiological stress during hospitalization, all of which may influence both glucose variability and hs-cTnT levels. Second, the assessment of glycemic variability was based exclusively on capillary glucose measurements obtained during hospitalization, typically over a short-term period and under intensive monitoring protocols, which may not accurately reflect patients’ long-term glycemic fluctuations in real-world settings. In addition, the retrospective reliance on electronic medical records may introduce information bias, including incomplete documentation, variability in measurement timing, and potential misclassification of comorbidities or medication exposure. Because inpatient glucose sampling intensity may vary across individuals and can influence variability metrics such as SD, we explicitly reported the distributions of both the number of capillary glucose measurements per patient and the length of hospital stay (Supplementary Table S1) to enhance transparency and contextualize the estimation of short-term glycemic variability. Moreover, mean inpatient glucose over the same monitoring window was not included in the multivariable models; therefore, we could not fully disentangle whether the observed association of glycemic variability with subclinical myocardial injury was independent of the overall inpatient glycemic level. Such measurement constraints could attenuate the applicability of the predictive model to ambulatory populations or to those with different monitoring frequencies. Future studies incorporating mean inpatient glucose (and ideally continuous glucose monitoring–derived metrics) alongside variability measures are warranted to better separate the effects of glycemic level from variability. Furthermore, because SMI was defined using the admission (baseline) hs-cTnT rather than serial troponin measurements, we were unable to characterize troponin trajectories during hospitalization or establish the temporal ordering between glycemic variability and myocardial injury; therefore, reverse causation cannot be excluded. In addition, because a non-diabetic comparator group was not included, we could not formally assess whether diabetes status modifies the associations observed in this study; future studies incorporating mixed populations and interaction analyses are needed to address potential effect modification by diabetes status.

Despite these limitations, the present study provides consistent evidence of an independent association between short-term glycemic variability and subclinical myocardial injury in hospitalized patients with type 2 diabetes. Moreover, the ROC and calibration results indicate that the proposed model can provide reasonably reliable individualized risk estimates within this inpatient setting, which may facilitate bedside risk communication and prioritization of further evaluation, pending external validation. Future research should aim to validate this model in multicenter, prospective cohorts, incorporate continuous glucose monitoring data to capture comprehensive glycemic patterns, and explore the potential impact of targeted interventions to mitigate glycemic variability on the prevention of silent myocardial injury in diverse diabetes populations.

## Data Availability

The original contributions presented in this study are included in this article/Supplementary material, further inquiries can be directed to the corresponding author.
