# Supplementary material for: Association of short-term glycemic variability with subclinical myocardial injury in hospitalized patients with type 2 diabetes: a retrospective cross-sectional study
Source: Front Med (Lausanne). 2026 Feb 10;13:1725076. doi: 10.3389/fmed.2026.1725076 (PMC12929514; doi:10.3389/fmed.2026.1725076)
Supplement: Supplementary file 1 [file Table_1.docx]

**Supplementary Table S1.** Distribution of inpatient glucose sampling intensity and length of stay in the study cohort (n = 324)

| **Variable** | **Median [IQR]** | **Range** |
| --- | --- | --- |
| Number of capillary glucose measurements per patient | 32 [24–41] | 18–56 |
| Length of stay, days | 6 [4–8] | 3–12 |
| Capillary glucose measurements per day | 5.4 [5.0–6.2] | — |

**Note:** Capillary glucose measurements were obtained as part of routine inpatient care, typically including pre-meal and bedtime measurements, with additional tests performed as clinically indicated.

**Supplementary Table S2.** Primary Admission Diagnoses for the Index Hospitalization (n = 324)

| **Primary admission diagnosis category** | **n (%)** |
| --- | --- |
| Diabetes-related management* | 142 (43.8) |
| Poor glycemic control without acute complications | 58 (17.9) |
| Acute infection (non-severe)** | 49 (15.1) |
| Diabetic microvascular complications | 36 (11.1) |
| Renal or metabolic disorders*** | 27 (8.3) |
| Other non-cardiac medical conditions | 12 (3.7) |
| **Total** | **324 (100.0)** |

**Note:** Diabetes-related management includes admission for optimization of glycemic control, insulin titration, or evaluation of chronic diabetes-related complications without acute cardiovascular events. Acute infections were predominantly mild to moderate in severity (e.g., urinary tract infection, respiratory tract infection) and did not meet criteria for sepsis or septic shock. Renal or metabolic disorders include electrolyte disturbances, diabetic nephropathy evaluation, or metabolic decompensation not requiring intensive care. Patients with acute coronary syndrome, acute heart failure exacerbation, myocarditis, or other overt acute cardiac conditions at admission were excluded according to predefined exclusion criteria.
